# 2686. High HIV and STI Rates in Patients with MPOX Identified in a Large National Database

**DOI:** 10.1093/ofid/ofad500.2297

**Published:** 2023-11-27

**Authors:** David Alfego, Suzanne Dale, Dusica Curanovic, Deborah Boles, Jonathan Williams, Ayla Harris, Thomas J Urban, Charles M Walworth

**Affiliations:** Labcorp, Burlington, North Carolina; Labcorp, Burlington, North Carolina; Labcorp, Burlington, North Carolina; Labcorp, Burlington, North Carolina; Labcorp, Burlington, North Carolina; Laboratory Corporation of America, Burlington, NC; Labcorp, Burlington, North Carolina; Monogram Biosciences/LabCorp, Lagun Beach, California

## Abstract

**Background:**

In mid-2022, mpox virus infection emerged outside of African countries to which it is endemic. Infections increased worldwide and were identified in 16 countries by late June 2022, with the US documenting the highest number of cases. Here we report patient characteristics and assess coinfection rates between those who tested positive for mpox and those who tested negative.

**Methods:**

On July 6, 2022, an FDA-approved PCR-based test for mpox (Orthopoxvirus) was launched with high-throughput modifications. The test is a qualitative assay that uses DNA extracted from swabs of the viral rash. Clinicians were instructed to swab or brush the base of the lesion for sample submission. Test volumes were evaluated, and mpox positivity rates were assessed by sex, age, and geography. Coinfection with HIV, HBV, HCV, and STIs was also assessed. Differences in coinfection rates were assessed for significance using chi square testing with Bonferroni correction for multiple comparisons.

**Results:**

Between July 6 and November 30, 2022, 48,818 samples were tested for 38,388 unique patients; 12,045 (24.7%) samples and 8,247 (16.9%) unique patients were positive for mpox. The median age of patients who tested positive was 35 years and 96.5% were male. Patients who were positive for mpox compared to those who were negative had significantly higher rates of coinfection with HIV (22.9% vs 6.0%, p< .0001), HBV (2.3% vs 1.6%, p< .005), chlamydia (10.4% vs 7.5%, p< .0001), gonorrhea (13.4% vs 7.3%, p< .0001), or syphilis exposure (11.0% vs 2.9%, p< .0001). In contrast, patients who tested negative for mpox compared to those with mpox had significantly higher rates of coinfection with HSV-1 (15.7% vs 1.7%, p< .0001), HSV-2 (10.5% vs 3.2%, p< .0001), VZV (14.1% vs 0.3%, p< .0001) and exposure to HCV (5.0% vs 4.7%, p< .0001). Testing rose rapidly, peaked by the end of July, and steadily declined by the end of November. Highest mpox positivity rates were seen in FL and GA, followed by NY, TX and CA.

**Conclusion:**

The rates of HIV, HBV, and most STIs were significantly higher among those positive for mpox; the rates of HSV-1, HSV-2, VZV, and HCV were significantly lower. These data also highlight the need for a comprehensive evaluation of all vesicular lesions for non-mpox etiology.

**Disclosures:**

**Dusica Curanovic, PhD**, Labcorp: employed by labcorp during study|Labcorp: Stocks/Bonds **Deborah Boles, PhD**, Laboratory Corporation of America: Stocks/Bonds

